# TLR8 agonist VTX-2337 enhances NKG2D-mediated cytotoxicity of NK cells

**DOI:** 10.1186/2051-1426-1-S1-P44

**Published:** 2013-11-07

**Authors:** Hailing Lu, Yi Yang, Veronika Groh, Thomas Spies, Gregory Dietsch, Maura Matthews, Mary L Disis, Robert Hershberg

**Affiliations:** 1 Department of Medicine, University of Washington, Seattle, WA, USA; 2 Clinical Research Division, Fred Hutchinson Cancer Research Center, Seattle, WA, USA; 3 VentiRx Pharmaceuticals, Seattle, WA, USA

## 

NK cells express an array of activating and inhibitory receptors, which facilitate the recognition and lysis of virally infected and transformed cells, but safeguard healthy cells from attack. NKG2D is an activating receptor expressed on the surface of NK cells that recognizes the ligands MICA/B and ULBP in human, which can be expressed on tumor cells or virus-infected cells. While NKG2D-mediated NK cell cytotoxicity plays an important role in tumor immune surveillance, cancer patients have been reported to have decreased expression and reduced function of the NKG2D receptor. We hypothesize that VTX-2337, a selective TLR8 agonist that activates myeloid DC and stimulates the production of pro-inflammatory cytokines including IL-12 and IL-18, may enhance NK cell function through NKG2D. PBMC from 11 donors were stimulated plate-bound anti-NKG2D antibody (clone 1D11), with or without prior incubation with VTX-2337 (500 nM, 24 hr). VTX-2337 significantly enhanced NKG2D-stimulated IFN-γ production and CD107a expression, as assessed by FACS. To further investigate the impact of TLR8 activation on NKG2D-mediated cytotoxicity, VTX-2337-treated PBMC were incubated with human lymphoblastoid cell line C1R transfected with the NKG2D ligand, MICA. Pre-activation of PBMCs with VTX-2337 enhanced the lysis of C1R-MICA cells. Collectively these data suggest that the TLR8 agonist VTX-2337 can enhance NK cell-mediated immune surveillance against cancer through NKG2D and the recognition of MICA.

